# Supplementary material for: Conditional chemoconnectomics (cCCTomics) as a strategy for efficient and conditional targeting of chemical transmission
Source: eLife. 2024 Apr 30;12:RP91927. doi: 10.7554/eLife.91927 (PMC11060718; doi:10.7554/eLife.91927)
Supplement: Figure 3—source data 3. [file elife-91927-fig3-data3.zip › Figure 3 source data3 part 2.zip!/Figure 3-Source data 3-part2/1062-13-CRISPResso_Report_jt28fhr9.zip!/CRISPResso_on_1062_15/1a.Read_barplot.pdf]

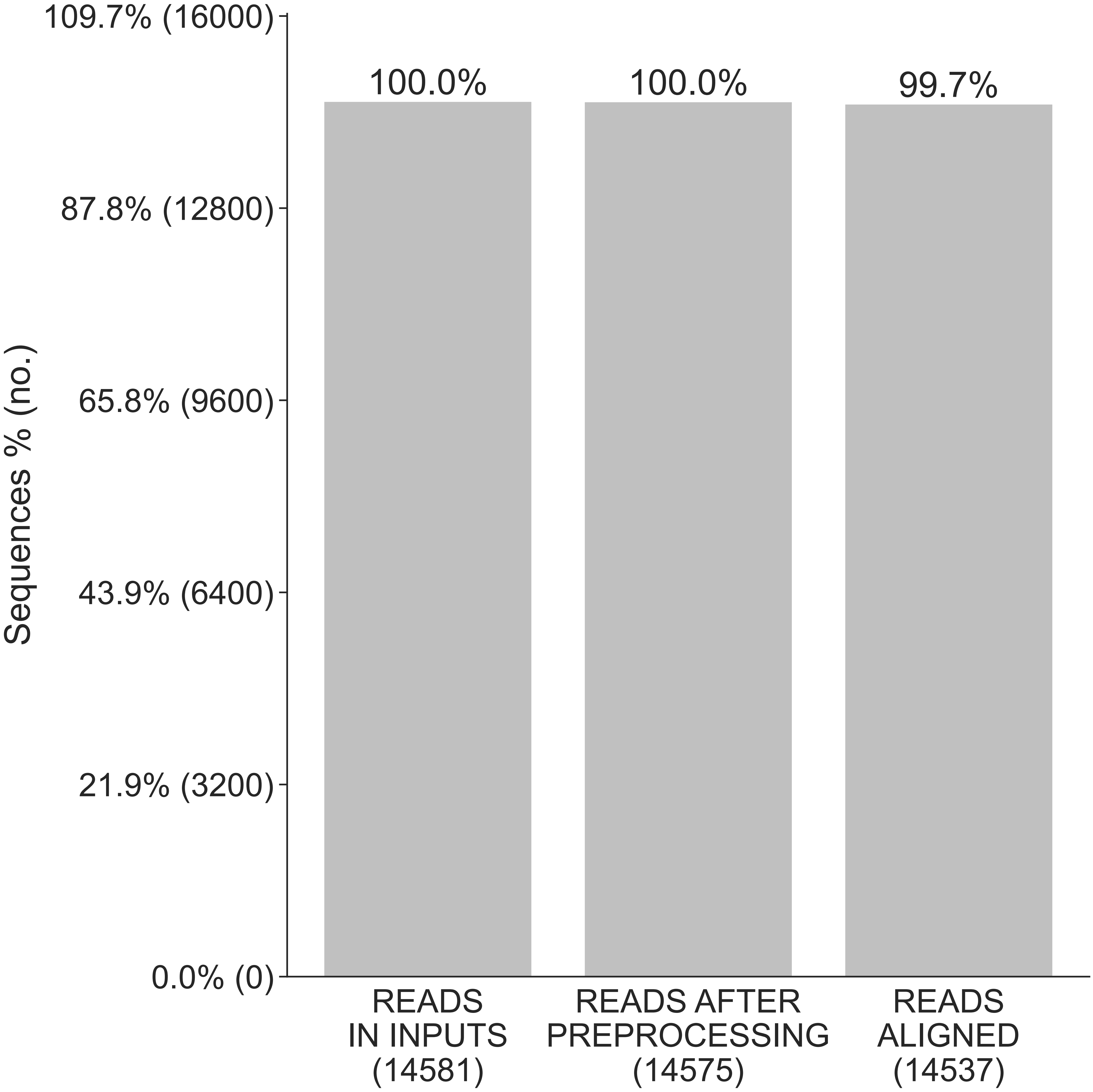
Sequences % (no.)
109.7% (16000)
87.8% (12800)
65.8% (9600)
43.9% (6400)
21.9% (3200)
0.0% (0)
100.0%
100.0%
99.7%
READS IN INPUTS (14581)
READS AFTER PREPROCESSING (14575)
READS ALIGNED (14537)
